# Editorial: Using gaze to study social knowledge: current challenges and future directions

**DOI:** 10.3389/fpsyg.2023.1225626

**Published:** 2023-06-13

**Authors:** Vanessa A. D. Wilson, Emily J. Bethell, Christian Nawroth

**Affiliations:** ^1^Department of Comparative Cognition, Institute of Biology, University of Neuchâtel, Neuchâtel, Switzerland; ^2^Department of Comparative Language Science, University of Zurich, Zurich, Switzerland; ^3^Center for the Interdisciplinary Study of Language Evolution (ISLE), University of Zurich, Zurich, Switzerland; ^4^Research Centre in Evolutionary Anthropology and Palaeoecology, Liverpool John Moores University, Liverpool, United Kingdom; ^5^Institute of Behavioural Physiology, Research Institute for Farm Animal Biology (FBN), Dummerstorf, Germany

**Keywords:** comparative cognition, developmental cognition, eye tracking, social gaze, gaze following, methodology, visual attention

Humans rely strongly on the visual modality for navigating their world, so it is no surprise that a focus on visual responses has become prevalent in the field of comparative cognition. This is especially relevant when studying cognitive processes amongst non-verbal subjects such as infants, but also for research on non-human animals. Such studies examine how participants inspect and discriminate stimuli, exhibit anticipation, visually respond to expectation violation (Tafreshi et al., 2014), or follow gaze (Shepherd, 2010). The goal of these approaches is to examine what information subjects attend to, and to draw inferences about physical and social knowledge in the studied individuals. Currently, however, methodological paradigms that rely on gaze are diverse and often provide varying definitions of measures or interpretations of findings (Haith, 1998; Winters et al., 2015). To strengthen the methodological approach of using gaze to measure cognition, this Research Topic has aimed to explore theoretical and empirical assessments of visual attention from a multidisciplinary perspective. It will focus in particular on the use of gaze in social cognition, since a comparative understanding of social knowledge is key to advancing theories of cognitive evolution.

In their review paper, Zeiträg et al. discuss different research methodologies, and recent advances, in the study of gaze following in human and non-human animals. Historically, a bias toward canids and primates limited evolutionary interpretations to only a few evolutionary lineages. This review gathers new insights from more recent studies across a broader range of taxa including fishes, reptiles, and birds. The authors propose that the foundations of gaze following emerged early in evolutionary history, such as the basic reflexive co-orienting seen in fishes (Leadner et al., 2021). This would provide a parsimonious explanation for the ubiquity of gaze following across amniotes. Subsequent evolution of more complex forms of gaze following, including the ability to form social predictions, may have co-evolved multiple times, resulting in part from increasing complexity of brain anatomy. Taxa in key phylogenetic positions are identified as targets to better understand the evolutionary history of social gaze following.

In a perspective article, Wilson et al. critically discuss the use of gaze studies in understanding cognitive processes in non-verbal individuals, particularly non-human animals. They highlight the limitations in interpreting data from paradigms that rely on looking time measures, and propose solutions to improve experimental approaches, considering the role of methodological validation, technological innovations and collaborative research in these solutions. Furthermore, they suggest considering gaze responses from an animal welfare perspective and advocate for implementing these proposals across the field of animal cognition and welfare to enhance experimental validity.

The remaining studies utilize eye tracking to address questions about engagement with social stimuli. Tomalski et al. examined changes in the complexity of visual scanning for infants at two developmental time points. For 5.5 and 11 month olds, they found more complex scan patterns to social compared with non-social scenes (both static and dynamic), including a higher number of recurrent fixations, repetition of fixation sequences, and longer fixation sequences to social stimuli. Moreover, this pattern increased with age, demonstrating an increased focus for social stimuli. Notably, results indicate a rather different pattern when examining number of fixations to stimuli, highlighting the importance of not only focusing on fixation length and occurrence, but also in examining sequential fixation patterns over time.

Champ et al. presented rhesus macaques (*Macaca mulatta*) with simulated dyadic social interactions depicting a dominance hierarchy, from which they extracted gaze patterns indicative of social engagement. They used these patterns to define differences in gaze following saccades and joint attention saccades. These saccades showed a higher frequency following a threatening or appeasing facial expression compared with neutral expression, and a higher number of joint attention saccades stemming from the subordinate toward the dominant monkey, rather than vice versa. This study demonstrates the potential application of combining dynamic stimuli and temporal scan patterns for understanding social cognition.

Dollion et al. present the first empirical eye-tracking study of whether the many benefits of service dogs to children with Autism Spectrum Disorder extend to improving decoding of facial expressions. Two groups of children, with or without a service dog, were tested on a facial expression recognition computer task and their ocular movements were tracked. Children who lived with a service dog exhibited more targeted face scanning of expression-relevant areas, and spent less time scanning areas of the face irrelevant for expression processing, although there was no difference between groups in accuracy for naming expressions of a response time. The authors suggest these results indicate that interacting with a service dog on a daily basis may enhance the development of visual scanning strategies for emotional face processing.

Finally, Sosnowski et al. used eye tracking to examine the impact of exogenous and endogenous oxytocin on visual attention to facial features in tufted capuchin monkeys (*Sapajus apella*). Their results suggest that oxytocin may not universally enhance eye contact and that endogenous and exogenous oxytocin may have different effects on social attention allocation. Specifically, frequency of looks to the eye region of faces increased following exogenous oxytocin administration (induced via fur-rubbing), but results were not consistent when examining gaze durations. In addition to highlighting differential effects of endogenous and exogenous oxytocin, this study highlights the importance of accounting for different gaze measures when drawing inferences on cognition.

Together, the articles in this Research Topic demonstrate both the multi-disciplinary application of gaze to understanding cognition, as well as the need for regular assessment of gaze-based methodologies that address reliability, validity, and data interpretation. The output of this Research Topic encourages not only methodological rigor and investment in novel techniques and approaches, but also emphasizes the applications of this research to fields such as animal welfare.

## Author contributions

All authors listed have made a substantial, direct, and intellectual contribution to the work and approved it for publication.
